# Collaborative Leadership, Collective Action, and Community Governance against Public Health Crises under Uncertainty: A Case Study of the Quanjingwan Community in China

**DOI:** 10.3390/ijerph18020598

**Published:** 2021-01-12

**Authors:** Qaunfeng Shu, Yahua Wang

**Affiliations:** 1 School of Public Policy and Management, Tsinghua University, Beijing 100084, China; sqf@mail.tsinghua.edu.cn; 2 China Institute for Rural Studies, Tsinghua University, Beijing 100084, China

**Keywords:** COVID-19, uncertainty, collaborative leadership, public governance, collective action

## Abstract

In the face of a public health crisis full of uncertainty, how should the community respond in order to effectively reduce the negative impact of the epidemic on public health? This article takes a Chinese rural community located near Wuhan City as an example to explore the mechanism of how collaborative leadership enhanced collective action in community governance against the COVID-19 pandemic. Early blockading to prevent transmission into the community, strict maintenance of social distance to prevent internal diffusion, timely elimination of public panic, and efficient guarantees of household supplies have proven effective in preventing the spread of the epidemic. Our research shows that collaborative leadership can achieve these goals mainly by effectively integrated local knowledge, modern information technology, and social self-organization, and then promoting the realization of collective action of community epidemic prevention and control. The lessons and implications for public health are discussed.

## 1. Introduction

The COVID-19 pandemic is a serious threat to human health and economic and social development as well as a great challenge to global leadership and national governance. The increasing diversity in influenza viruses, which suggests the possibility of further respiratory pandemics, has shown a clear acceleration in global variation and epidemic frequency, and now significantly exceeds that in the 20th century [1]. At the same time, the epidemic has also uncovered another important insight: the world exists as a risk society [2]. A risk society is based on the modern transformation of industrial society, which means multiple uncertainties that may bring uncontrollable risks or disasters. Particularly, with respect to sudden public health crises, it often brings to people not only fear or sadness, but also great confusion, because people face an unprepared environment and unknown consequences at any time. Therefore, in an era full of uncertainty, the decisions made by organizations at different levels and the management styles they show will affect our economic, political, and cultural governance and lifestyle, and even the appearance of a region, a country, or the world itself. This is a warning that in the era of globalization, each country or region must be ready to deal with biological threats such as infectious diseases, and constantly improve its leadership and decisions regarding public health crises.

As the area in which the outbreak first occurred, China was the first to control the epidemic. Despite the second wave of small-scale outbreaks in Heilongjiang Province and Beijing City, COVID-19 was still quickly controlled. One key to this success was effective isolation at the community level, which ensured that infectious sources would not spread within the community, and additionally blocked importation of the infection from outside the community. Although rapid detection and infection path tracking have been proven to be effective, it is undeniable that its effectiveness is still based on a certain degree of community blockade and home isolation, buying enough time for effective medical intervention. Especially in areas with relatively underdeveloped economy and medicine such as rural communities in China or other developing countries, rapid and effective response at the community level is particularly important. Thus, community capacity building is very important when faced with unknown public health emergencies. Thus, how can Chinese rural communities, which have few facilities and resources, successfully cope with the COVID-19 pandemic? What are the key factors and paths for its success? What kind of enlightenment can it bring to the promotion of public health? Based on a case study of a rural community in China, this study explores, from the perspective of leadership, how the rural community promoted successful collective action in the early, highly uncertain stages of the COVID-19 pandemic.

## 2. Literature Review

### 2.1. Collective Action and Changing Leadership Needs

Since the dawn of human civilization, collective action has been used in societies throughout the world and the causes and mechanisms of this phenomenon are fascinating to researchers [3]. For Charles Tilly, a famous sociologist in the United States, collective action meant people acting together to pursue their common interests [4]. In the global public health crisis represented by COVID-19, both government officials and the general public, in both developed and less developed countries, need to take effective collective action to protect public health. The question is how to achieve successful collective action in public health crisis governance. Leadership is critical for successful community cooperation [5], and leaders are regarded as indispensable to improving public performance and promoting organizational change [6]. People have long recognized that to do anything well in a community, there must be leaders willing to take charge to help turn their vision into action [7,8,9].

With the advent of globalization and risk society, the environment of public governance is becoming more and more complex and demanding. Public managers need to adopt new leadership behaviors to realize the full value of public services to serve the interests of citizens [10]. The mechanisms of a community are typically highly autonomous. However, different groups in the community such as leaders, teachers, and doctors, the rich and the poor have complex relationships with each other. In this kind of partnership, leaders often lack strict control over community members and their actions [7]. Whether in a democracy or an authoritarian regime, it is difficult for leaders of the old “command and control” style to survive in today’s highly interconnected and cooperative environment and adapt to the complex governance situation [11]. Therefore, new leadership behaviors are urgently needed.

China is a typical authoritarian country [12], and its public sector often follows the traditional leadership style, represented by top-down bureaucratic control. The role of a leader is often manifested in the form of rigid command, order, and task arrangement, which overemphasizes the authority of the leader and ignores the interaction with employees and other organizations, that is, a type of controlled leadership based on power. Conversely, the governance of a public health crisis such as COVID-19 involves multiple actors including leaders, the public, and experts, and multiple concerns such as maintaining social distance, isolating possibly infected persons, and ensuring household supplies: only if all stakeholders cooperate in making decisions and taking collective action against the epidemic can we ensure the coordination of as many resources as possible and mobilize as many people as possible to participate. These include both public sector employees and a large number of private sector employees, which renders the traditional controlled leadership ineffective and makes collaborative leadership an important component of public health crisis leadership [13,14,15]. 

### 2.2. Collaborative Leadership and Public Health Crisis Response

Collaborative leadership is leadership that is collaborative. This definition refers to taking a leadership role in a coalition, organization, or other enterprise where everyone is on an equal footing and works together to solve a problem, create something new, or run an organization or initiative [16,17]. The leader is not in control of the group, but rather has the responsibility for guiding and coordinating the process by which the group decides upon and carries out actions to accomplish its goals [18]. People often find collaborative leadership particularly useful in situations where there are issues or problems so complex that no one person or entity has either the information or the power to change them [19]. 

Gostin and Friedman indicated that the global spread of the Ebola virus reflected a failure of global health leadership; it was not only a public health crisis, but also a collaborative effort crisis [20]. Leaders have increasingly realized that the level of leadership plays an important role in whether they can successfully survive public health crises such as the Ebola epidemic. In particular, the global pandemic of COVID-19 has reminded the world of the highly significant and urgent need for quality leadership [21].

Since December 2020, the global public health risk has become increasingly serious. However, when aspiring leaders are ready to deal with it, they often lack effective experience and effective models or frameworks to guide them [22]. As public health is “public”, leaders are required to perform on stage in full view of the world [23]. Particularly in public health crises, media attention often aggravates the tension of public health issues and complicates political relations. Due to the extensive and interdisciplinary nature of this field, leaders must carefully build alliances as wide-ranging as possible, unite potential partners who are not willing to cooperate, and coordinate different views, goals, and resources [24]. Therefore, leaders in public health come from not only from within the profession, but also, and perhaps more importantly, from outside it [25].

Collaboration is a higher level of collective action than cooperation and coordination [26], in which autonomous participants identify or resolve their common concerns through formal or informal negotiations, jointly formulated rules, and organizational structures. Effective epidemic prevention and control requires the combined and the coordinated information, resources, activities, and capabilities of many organizations and groups; thus, it cannot be achieved by a single organization or group [27]. At such times, the efforts of collaborative problem-solving and decision-making that engage people constructively across the boundaries of public agencies, levels of government, and/or the public, private, and civic spheres will be crucial [28] to gather all the internal and external forces in the field of public health through effective communication and decision-making [29]. Local leadership rooted in the community, especially collaborative leadership, can effectively mobilize the community in public health crisis governance to promote successful collective action against the epidemic [3].

## 3. Research Methods

### 3.1. Qualitative Case Study

This study used a qualitative case study as the main research method, with participant observations and unstructured interviews as the main data collecting method. Deep description was the main narrative means. The selection of the research method depends on the purpose of research, the nature of the issues under study, and the availability of research data, among other factors. Qualitative research aiming at exploration is quite applicable to an in-depth study of complicated matters and processes, unknown phenomena, and cases where experiments are impossible for practical reasons [30,31]. As this study attempts to describe the process of different organizations and individuals in the community participating in the prevention and control of the COVID-19 epidemic, and explore the impact and path of collaborative leadership on community collective action against the epidemic, the qualitative research method is thus appropriate and scientific.

### 3.2. Case Selection and Sources

**Figure 1:**
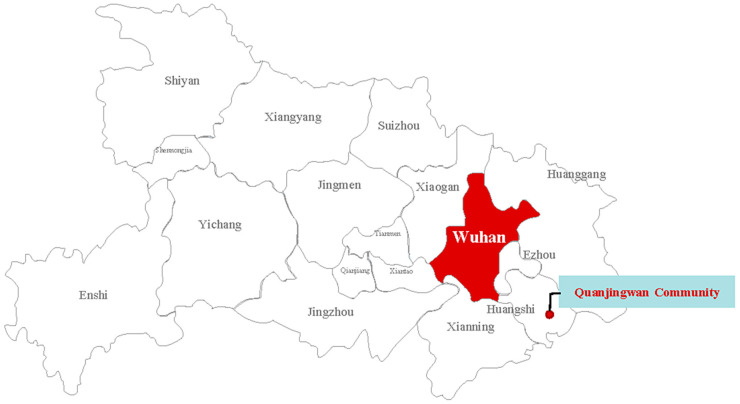
The geographical position of Quanjingwan community in Hubei Province, China.

This article offers a case study of the practices of a rural Chinese community, Quanjingwan, in Yangxin County, Hubei Province. Quanjingwan community is approximately 200 km from Wuhan, the epicenter of China’s epidemic (Figure 1). Quanjingwan is a village with a traditional clan culture. It has 118 households with a total of 765 registered residents, and there were 85 households with 536 people left in the village before the closure of the community. Among them were 63 migrant workers who had recently returned from Wuhan. These were generally considered as likely to be infected with the virus. Quanjingwan was closed on 24 January and reopened on March 14. There were three reasons for choosing the Quanjingwan community as the sample of a single case study. First, the Quanjingwan Community is a representative village. Since the beginning of the 21st century, the widespread decline in the rural population worldwide has diminished rural public governance, reshaped the rural social structure and governance form, and directly affected the governance ability of rural communities in terms of infrastructure, public services, public health, and other public goods supply. Quanjingwan is a rural community with a population loss rate of approximately 30%. Thus, the study of its performance and ability in coping with a public health crisis would be significant. Second, the Quanjingwan community has certain remarkable characteristics. It is very close to the epidemic center, and the number of people returning from Wuhan is large. However, because of the anti-epidemic collective action taken by the villagers, the number of confirmed or suspected cases in the Quanjingwan community was zero, while there were 10 cases in the adjacent village. Cooperation between agents and across organizations laid the foundation for the success of collective action. Third, one of the authors was living in the Quanjingwan community during the outbreak of COVID-19, and the author participated in, observed, and recorded the whole anti-epidemic process in the village, which proved to be a boon as the author was able to obtain valuable first-hand survey data and information for the case study.

## 4. Case Analysis: Collaborative Efforts in the Community against the Epidemic 

Regardless of whether a state’s government is strong or weak, it must work with citizens to solve problems [17]. As the most basic unit of governance, the community is the first line of epidemic prevention and control, and has the responsibility of preventing infection from both external and internal spread. At an early stage of the epidemic, the prevention and control of the epidemic in Quanjingwan community faced great uncertainty. First, the full picture of the epidemic crisis was unclear: at the beginning of the epidemic, both scientists and government officials only knew that it was an infectious disease similar to Severe Acute Respiratory Syndrome (SARS), and its transmission path and diffusion law were not clear. Therefore, the 63 migrant workers who returned from Wuhan after 1 January 2020 were like a time bomb, causing widespread panic among the villagers. Second, the duration of the crisis could not be predicted; even the authoritative medical experts and researchers such as academician Zhong Nanshan, who is a well-known figure associated with the SARS epidemic, could only preliminarily predict the development in the short-term, and additionally, the prediction of peak value, inflection point, and duration changed dynamically with the situation, which increased the uncertainty. Third, there was a serious shortage of medical resources, especially masks. Masks are considered the simplest but most effective way to block the spread of the epidemic. However, there was a great shortage of masks in China at that time, and it was even more difficult to purchase masks in rural areas. Once the epidemic spread widely in rural areas, it would inevitably create a disastrous public health crisis. 

In the face of multiple uncertainties, the leadership behavior of Quanjingwan community became particularly important. Quanjingwan community was taken as an example to analyze the form of collaborative leadership in anti-epidemic activities and examine how to promote collective action.

### 4.1. Blockading the Community: Sharing Control between Organizations

The epidemic started before the Spring Festival, the most important festival in China. It is traditionally a time for visiting relatives and friends, and it is the time of year when population flow is the largest and personal contact is the most frequent. With the blockade of Wuhan City and Yangxin County, it became urgent for Quanjingwan to prevent the virus from entering the community.

**Figure 2:**
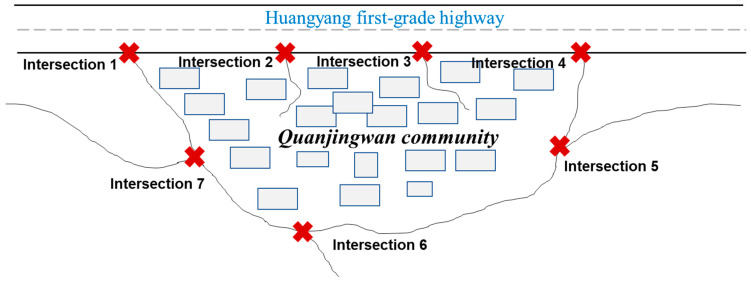
Road intersections around Quanjingwan.

At approximately 3 p.m. on 24 January 2020, the leader of the community (the director of the village committee) planned to blockade the community on the advice of the community doctor. There are seven road intersections around Quanjingwan (Figure 2), and as the village committee has only four members, they could not prevent people outside from entering the village through the intersections. Quanjingwan also has a council of rural sages (CRS), who are informally elected by the residents. These are mainly clan elders, economic elites, and retired cadres who live in the village. They have a high level of public service motivation and leadership as they often provide voluntary services to the villagers and enjoy high prestige and trust. For example, they donate money for the construction of public facilities in the village, take the initiative in caring for lonely elderly persons in the village, and lead and organize the villagers to maintain the damaged rural roads or canals together. Although the members of the village committee and the CRS are elected by the villagers, to a certain extent, the village committee represents the interests of the government, while the CRS fully represents the interests of the villagers. The villagers trust the CRS more than the village committee and often participate in the activities organized by the CRS, while they do not participate in the activities organized by the village committee. Therefore, there are occasional power conflicts between the CRS and the village committee. The director of the village committee has a certain hostility toward the CRS, and thinks that the CRS weakens the power of the village committee.

However, in the face of a critical situation and a lack of human resources, the director of the village committee realized that he had to face and mediate the conflict directly, and furthermore, that a change in leadership style had to be made: partners must be trusted and valued, differences overlooked, and control shared [32]. The director of the village committee established a chat group through WeChat, a social software in China, and invited two village cadres and two leaders of the CRS to join the WeChat group. The director of the village committee publicly expressed his apology to the director of CRS in the WeChat group, and hoped that everyone would abandon their previous suspicions and reach consensus to deal with the public health crisis. After discussions, the leaders of the two sides agreed that the village committee and the CRS should be cooperative rather than competitive. Their organizational goals and principles were the same, namely, they were committed to maximizing the public interest of the community residents, and in the face of an epidemic threatening the health of residents, they needed to cooperate and act together. Soon, the CRS quickly gathered seven members and 10 resident volunteers and completed the community blockade with the village cadres within two hours. To do so, they blocked the roads into the village with mounds of earth or stones so that cars could not go in or out, and they stationed people at each intersection to prevent non-residents from walking in.

In conjunction with preventing outsiders (and possibly viruses) from entering the community, the director of the village committee helped the community doctor start screening and registering those returned from Wuhan. As per the government’s instructions, people who had been in Wuhan in the last 14 days were to self-isolate at home for 14 days, and the doctor took their temperature every day to check for symptoms of COVID-19. They again consulted with the CRS and asked them to find volunteers to purchase household supplies for the quarantined persons and dispose of their household garbage. Forbidding travel and isolating people at home not only prevented infected people from bringing disease into the village from the outside, but also reduced the chance that it could spread within the village.

### 4.2. Keeping Social Distance: Coordinating the Function of Autonomy and Regulation

As part of the strong traditional culture in rural China, cultural activities, banquets, and card games (for both entertainment and gambling) are constantly occurring. These are traditional activities that have been passed down for thousands of years; however, they all involve gatherings of people, which would provide a great opportunity for COVID-19 to spread. It is not difficult to prohibit large public events like cultural activities; however, the same could not be said of controlling private gatherings like banquets or card games.

China’s rural communities are legally autonomous, so it would be hard for the village committee, the leading body of community, to prevent such gatherings. The community leaders decided to cooperate with the Council of Weddings and Funerals (CWF), another community organization, to help prevent the epidemic. In China’s rural areas, marriages and funerals can be major events for the whole village, with many intricate steps: all villagers participate, either in the ceremony itself or in the preparations. The CWF coordinates all of this work. Like the CRS, the CWF is completely independent of the village committee and it is usually composed of the older villagers and those who have received a traditional cultural education. They have experience and prestige, they are very familiar with traditional wedding and funeral culture and etiquette, and the villagers usually trust them very much.

Under the common appeal of the village committee and the CWF, many of the planned banquets in Quanjingwan were cancelled. However, it was hard to prevent small, quiet gatherings. Early in the blockade, when the villagers had little awareness of the risk, some still secretly played cards or mahjong. Community leaders had two responses. First, they used loudspeakers throughout the day to repeatedly publicize the strong diffusivity and susceptibility to infection of the novel coronavirus and encouraged community solidarity [33]. Second, they asked the police to punish villagers who gathered to gamble, in accordance with the Regulations on the Administration of Social Security. This combination of responses essentially succeeded in controlling social gatherings in Quanjingwan.

### 4.3. Easing the Panic: Mobilizing Intellectual Elites to Exert Their Strengths

After two weeks of community blockade, the epidemic situation across Yangxin County had not improved. As there was no clear official news, an increasing number of villagers became depressed or upset. Internet rumors spread among the villagers, inciting panic. Due to the imbalance of urban and rural development in China, those who could master capital, technology, knowledge, and other resources left the countryside when they were still young in order to seek jobs in the city; these are often the elites of the village. Those left in the countryside including the community leaders were often older, and knew little new about information technologies such as the Internet and online social networks. Therefore, it was hard for the community leaders to know how to alleviate the panic caused by online networks.

Realizing their own limitations, the leaders of the village committee encouraged some intellectuals such as schoolteachers and college students who were proficient in Internet technology to rapidly refute false information, usually through the community’s WeChat group, and forward the latest epidemic information (from the official media) to the group. These intellectuals also looked for helpful information on the Internet that the community leaders could use, for example, they proposed changing the roadblocks from heaps of rocks to movable branches or railings in order to allow emergency car trips from the village to the hospital, and to allow essential government vehicles to enter.

The Internet Age is an era of information explosion. It is a challenge for the general public to identify accurate and effective information. The intellectuals in the community used their Internet expertise to identify false rumors, gather authoritative information, publicize official policies, purchase epidemic prevention materials online, and obtain online psychological counseling for villagers. Thus, they fostered collective action in public health crisis governance and promoted effective collaboration among different groups [34] including voluntary compliance with the government’s epidemic prevention policy.

### 4.4. Guaranteeing Household Supplies: Encouraging Economic Elites to Contribute

Epidemic prevention and control is a long-term process, and, although the measures will seriously harm economic development, they are required for public health. Of course, this kind of exchange is only worthwhile if the social order can be maintained. 

The key to ensuring that stability is to ensure the reliable provision of household staples. As the blockade continued, the villagers’ supplies ran low. A survey of the community found that 70% of the families did not have enough rice, flour, and other food for another 20 days, but due to the blockade, it was not convenient for them to go out to buy food.

In recent years, some economic elites from Quanjingwan who did business in the city returned to their hometown to start new businesses such as farms, or contracted for large tracts of cultivated land. In the context of rural China, if such entrepreneurship is supported by community leaders, the business would be helped, and if it is also recognized by community residents, the sense of achievement among the economic elites would greatly improve. In Quanjingwan, community leaders encouraged wealthy entrepreneurs in the community to apply to the government for vehicle passes and to purchase essential goods for residents every two days. However, due to the size of the community, the burden of these large purchases on the entrepreneurs was large, causing mistakes, and thus complaints from the villagers.

**Figure 3:**
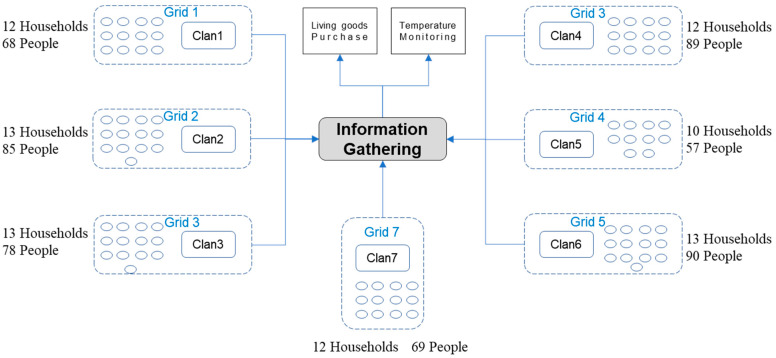
Distribution of Quanjingwan community grids based on clanship.

The head of the CRS suggested dividing the village into seven grids based on clanship. Households in each grid mostly belong to the same clan (Figure 3), so their internal communication channels are unblocked and the communication cost is very low. Each grid would elect one member as its representative to tally up the household goods needed and to help the community doctor monitor the residents’ temperatures. This measure greatly reduced the pressure on both the entrepreneurs and the doctor, and greatly improved the satisfaction and cooperation of the villagers. The ban on travel, even on foot, was complied with, constituting an example of effective collective action to fight the epidemic.

## 5. Discussion

**Figure 4:**
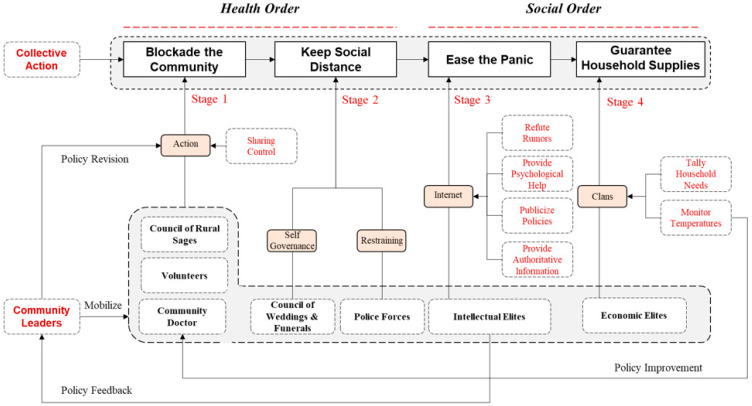
Collaborative efforts in community anti-epidemic actions in Quanjingwan.

Taking the Quanjingwan community as a case study, we studied how collaborative leadership played a role in the collective action against the COVID-19 pandemic in rural China. Some lessons can be learned from this case. Given the complexity and uncertainty of public health events and the multi-agent characteristics involved in public health crisis governance (Figure 4), it is necessary to attach great importance to the leadership role of the community, especially to utilize the new leadership rooted in community environment and culture [8,35]. In community-level anti-epidemic action, an effective health order is needed, but so too is a stable social order. The establishment of these two kinds of order requires the participation of all community members such as maintaining social distance, avoiding travel, and so on. The anti-epidemic practice of Quanjingwan provides valuable lessons and implications for public health.

Viruses are the common enemy of all mankind. The accelerated spread of the COVID-19 pandemic concerns the whole world, and not only has it promoted fear and worry, but has also sounded an alarm for those who do not wish to strengthen global cooperation. In fact, responding to COVID-19 and protecting public health is not only a matter of the natural sciences such as medicine and pharmacy. As a public good, public health, in a modern society full of uncertainty, of course, needs the involvement of social sciences, especially those that inspire collective action and public governance. Martin Alblau, a well-known British sociologist stated that COVID-19 and other major public health crises need the collective wisdom and the collaboration of all mankind [36]. This communal wisdom includes not only modern information technology with universality, but also local knowledge with specific scope. The case of Quanjingwan demonstrated that local knowledge rooted in community history and tradition including clan culture can reduce the uncertainty of new governance and bridge the gap between theory and practice. Meanwhile, modern information technology such as online social networks can aid individual decision-making [37], streamline the operation of organizations, reduce the cost of coordination and crisis communication [38], reduce the negative impact of uncertainty on public behavior and psychology, and improve the efficiency of the organization and mobilization of collective action [39]. Local knowledge, modern information technologies, and social self-organizations will play an important role in helping the community to cope with the public health crisis and ensure public health in a low-cost, efficient, and rapid way.

The case study of a rural Chinese village caught in the global epidemic dynamics of 2020 demonstrates that, in the face of uncertain public health crisis, effective cooperation and collective action are the only ways to enact successful anti-epidemic tactics. Therefore, the development of collaborative leadership has become particularly important. Traditional leadership is oriented to achieve organizational goals, while collaborative leadership takes solving public problems as its value pursuit; traditional leadership uses authority to motivate followers through organizational and bureaucratic ways; collaborative leadership emphasizes “shared power” or power sharing, rather than authority control through partnership and mutual learning [6,40]. Evidently, in order to achieve effective cooperation, the importance of collaborative leadership in public governance must be emphasized and celebrated because it can reduce transaction costs, enhance social norms, promote social trust, and then promote the achievement of collective action [41]. The collective action in Quanjingwan demonstrates effective instruments to use to promote cross-sector and cross-subject cooperation, which lays a foundation for successful anti-epidemic actions at the community level. At the same time, it shows us that, not only at the community level, but also at the regional level and even at the national level, to provide support for good governance, it is necessary to prioritize and improve collaborative leadership. In fact, the world will be in a risk society for a long time, and will inevitably encounter more and more public crises full of uncertainty such as public health crises, climate change crises, environmental governance crises, and so on. In order to manage these public crises, effective collective action is required, and furthermore, developing and adopting collaborative leadership will be the key factor.

## 6. Conclusions

This article presents a glimpse of how collaborative leadership can support successful community governance against the public health crisis of COVID-19. Facing such a threat, all organizations and individuals in the community, whether at the global, national, regional, or grass-roots level, have the obligation to actively participate in collective action through cooperation. The case of Quanjingwan shows that the spread of the virus can be prevented through effective collective action supported by partnerships in the community, and the key to realize such a success is through the collaborative effort of different organizations and individuals. According to the real time statistics of Johns Hopkins University, by the end of December 2020, more than 80 million people in the world had been infected, and nearly 1.8 million people had lost their lives. This is a public health tragedy. Even in China, where the epidemic was under initial control, scientists warn that China still faces the risk of another COVID-19 wave [42]. 

In the face of public health crisis with limited information, regardless of whether in poor countries or rich countries, democratic countries, or authoritarian countries, it is necessary and effective to take emergency public health interventions like community blockade. For example, a team of scientists from China, the United States, and the United Kingdom estimated that after the outbreak, a series of public health (non-drug) interventions in the first 50 days such as community blockade and public gathering prohibition reduced the total number of patients in China by more than 700,000 [43]. Although it will inevitably bring temporary economic and employment losses, health and life should be regarded as the priority of all human behaviors. When a community or a city needs to be blocked due to the impact of the epidemic, the effective collaboration among official organizations, autonomous organizations, and ordinary citizens is particularly important. As shown in this study, collaboration can generate mutual help behavior in the community, thus improving the individual and community’s ability and time to bear economic and employment losses to gain more time for epidemic control and medical progress such as the development of effective vaccines.

In summary, in response to a public health crisis like the COVID-19 pandemic, we should prioritize and enhance collaborative leadership in public governance and respect the positive roles of local knowledge, modern information technology, and social self-organization. In the face of the COVID-19 crisis and despite the uncertainty, early community blockading, strict maintenance of social distance, timely easing of public panic, and efficient household support can enable the people in a community to cope with the new challenges and ensure their continued life and health through effective collective action. In order to face such a serious public health crisis like the COVID-19 pandemic, all countries in the world must unite and stand together. We believe that this case study has certain reference value for community epidemic prevention and control in other underdeveloped and densely populated areas in the world, especially in South Asia, Africa, and Latin America.

The theoretical contribution of this paper is two-fold. On the one hand, it deepens the theoretical cognition of the relationship between leadership and collective action, unearths the important role of collaborative leadership in public health crisis response, and contributes a new variable and perspective to the theoretical research on collective action. On the other hand, it explores the specific mechanism of collaborative leadership that influences collective action. Although the importance of collaborative leadership in the field of public health has been widely recognized, few studies have explored why and how collaborative leadership can promote inter-departmental, inter-organizational, and inter-individual cooperation to achieve effective collective action to ensure public health. On the basis of the classic theory of collective action, this study attempted to use a single case study to fill this gap. In other words, collaborative leadership takes solving public problems as its value pursuit and emphasizes power sharing, integrating all kinds of human resources, information, and local knowledge to achieve effective collective action through reducing transaction costs, enhancing social norms, and promoting social trust. However, this study has some limitations. In the canons of social science research, a single case study, when compared with quantitative forms of research or multiple case studies, is ordinarily judged to be lacking in rigor, comparability, and replicability. Although the anti-epidemic experience of the Quanjingwan community can bring some positive enlightenment to the public health response under conditions of uncertainty, more effective, comparative, cross-cultural, cross-regional, and cross-country studies are needed to determine whether these experiences can withstand the test of different virus types, social and cultural situations, and political systems.

## Data Availability

No new data were created or analyzed in this study. Data sharing is not applicable to this article.
